# The Association between Maternal 25-Hydroxyvitamin D Concentration during Gestation and Early Childhood Cardio-metabolic Outcomes: Is There Interaction with Pre-Pregnancy BMI?

**DOI:** 10.1371/journal.pone.0133313

**Published:** 2015-08-05

**Authors:** E. Jessica Hrudey, Rebecca M. Reynolds, Adriëtte J. J. M. Oostvogels, Ingeborg A. Brouwer, Tanja G. M. Vrijkotte

**Affiliations:** 1 Department of Public Health, Academic Medical Center, University of Amsterdam, 1100 DD Amsterdam, The Netherlands; 2 BHF Centre for Cardiovascular Science, Queen’s Medical Research Institute, University of Edinburgh, Edinburgh, United Kingdom; 3 Department of Health Sciences, VU University, de Boelelaan 1085, 1081HV, Amsterdam, The Netherlands; University of Missouri, UNITED STATES

## Abstract

Both maternal 25-hydroxyvitamin D(25OHD) status and pre-pregnancy BMI(pBMI) may influence offspring cardio-metabolic outcomes. Lower 25OHD concentrations have been observed in women with both low and high pBMIs, but the combined influence of pBMI and 25OHD on offspring cardio-metabolic outcomes is unknown. Therefore, this study investigated the role of pBMI in the association between maternal 25OHD concentration and cardio-metabolic outcomes in 5-6 year old children. Data were obtained from the ABCD cohort study and 1882 mother-child pairs were included. The offspring outcomes investigated were systolic and diastolic blood pressure, heart rate, BMI, body fat percentage(%BF), waist-to-height ratio, total cholesterol, LDL cholesterol, HDL cholesterol, triglycerides, glucose, C-peptide, and insulin resistance(HOMA2-IR). 62% of the C-peptide samples were below the detection limit and were thus imputed using survival analysis. Models were corrected for maternal and offspring covariates and tested for interaction with pBMI. Interaction with pBMI was observed in the associations with insulin resistance markers: in offspring of overweight mothers(≥25.0kg/m^2^), a 10 nmol/L increase in maternal 25OHD was associated with a 0.007(99%CI:-0.01,-0.001) nmol/L decrease in C-peptide and a 0.02(99%CI:-0.03,-0.004) decrease in HOMA2-IR. When only non-imputed data were analyzed, there was a trend for interaction in the relationship but the results lost significance. Interaction with pBMI was not observed for the other outcomes. A 10 nmol/L increase in maternal 25OHD was significantly associated with a 0.13%(99%CI:-0.3,-0.003) decrease in %BF after correction for maternal and child covariates. Thus, intrauterine exposure to both low 25OHD and maternal overweight may be associated with increased insulin resistance in offspring, while exposure to low 25OHD in utero may be associated with increased offspring %BF with no interactive effects from pBMI. Due to the limitations of this study, these results are not conclusive, however the observations of this study pose important research questions for future studies to investigate.

## Introduction

Exposure to certain nutritional factors in utero, such as insufficient maternal 25-hydroxyvitamin D (25OHD), may be related to adverse cardio-metabolic outcomes in offspring [1, 2]. Maternal 25OHD deficiency can occur during pregnancy, in part as a result of fetal demand, and the prevalence varies from 5 to 67% depending on location, ethnicity, and definition of deficiency [2–4]. Laboratory and observational evidence suggest that an individual’s own 25OHD status may influence the risk for developing chronic diseases, such as type 2 diabetes mellitus and cardiovascular disease [3, 5] and some studies suggest that maternal 25OHD status during gestation also influences this risk in offspring [2, 6, 7]. Therefore it is possible that maternal 25OHD deficiency during gestation contributes to cardio-metabolic abnormalities in offspring, which then track into adulthood and increase the risk of future chronic disease [2, 3, 8].

Inconsistencies exist in the current literature on maternal 25OHD and offspring cardio-metabolic outcomes. Low maternal 25OHD has been associated with insulin resistance and with increased fat mass in young children [4, 9], while other studies have not observed these relationships [10, 11]. Therefore the relationship between maternal 25OHD status and offspring cardio-metabolic outcomes warrants further investigation.

In addition to maternal 25OHD status, pre-pregnancy BMI (pBMI) is also related to fetal development. Underweight increases the risk of low 25OHD levels in both pregnant and non-pregnant women [12, 13], and in pregnant women, it increases the risk of intrauterine growth restriction [14]. Overweight is also associated with low 25OHD levels in both pregnant and non-pregnant individuals [13, 15], and obesity during gestation is associated with offspring cardio-metabolic abnormalities [16–18]. Additionally, higher pBMIs are associated with lower 25OHD concentrations in neonatal cord blood indicating that less 25OHD reaches a fetus if the mother is obese [19, 20].

In the current literature on 25OHD and offspring cardio-metabolic outcomes, limited attention has been given to the role of pBMI and its potentially non-linear relationship with 25OHD. To the best of our knowledge, no study has investigated interaction with pBMI in these relationships. Therefore, we aimed to clarify the role of pBMI, as well as address previous inconsistencies, by testing interaction with pBMI in the association between maternal 25OHD during gestation and a range of cardio-metabolic outcomes in five to six year old children. We hypothesized that the combination of low maternal 25OHD during gestation and abnormal pBMI would contribute to greater abnormalities in early childhood cardio-metabolic outcomes than would intrauterine exposure to either of these maternal variables separately.

## Materials and Methods

### Study Design

**Fig 1:**
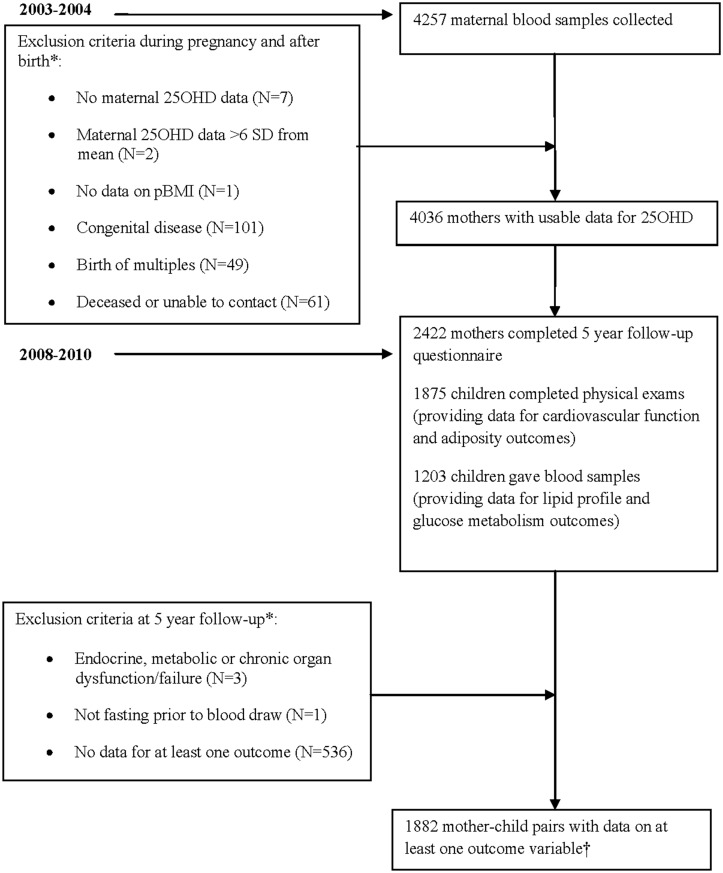
Flow chart of study participants. Selection of study sample from data collected for the Amsterdam Born Children and their Development cohort study. 25OHD = 25-hydroxyvitamin D, pBMI = pre-pregnancy BMI, SD = standard deviation. *Note: Exclusion criteria overlap in some participants. †Note: In the final study sample, some children completed only the physical exam and some only provided blood samples. As a result the final sample size is greater than the number of children measured in just the physical exam or just the blood testing component.

Data were derived from the Amsterdam Born Children and their Development (ABCD) study, a prospective observational cohort study that began in 2003 [21]. Between January 2003 and March 2004, all pregnant women in Amsterdam were invited to complete a questionnaire and volunteer a blood sample during their first prenatal screening. Mothers were contacted for follow-up when the child from this pregnancy reached five years of age. The participating children underwent one-day physical examinations and provided blood samples via finger prick at their schools or at local museums in Amsterdam. Maternal exclusion criteria included lack of data on 25OHD (N = 7) and pBMI (N = 1), and extreme 25OHD values (more than six standard deviations from the mean) (N = 2). Offspring exclusion criteria included twins or multiples (N = 49), non-fasting children (N = 1), congenital disease (N = 101), metabolic diseases which included cystic fibrosis, type 1 diabetes mellitus and genetic metabolic disorders such as familial hypercholesterolemia (N = 3), and lack of data on at least one outcome variable (N = 536). The final study sample consisted of 1882 mother-child pairs (Fig 1), which was greater than [4, 9, 10] or comparable [11] to the sample sizes of previous studies on this topic. Written informed consent was obtained via questionnaire from the participating pregnant women. At this time, women were also asked for written consent for future follow-up. Of those mothers who gave written informed consent for follow-up, parents and/or guardians gave consent via questionnaire for their five to six year old child to take part in the health check. The ABCD study and its informed consent procedure were approved by the medical ethics review committees of the Academic Medical Centre, Amsterdam and the VU University Medical Centre, Amsterdam, by the Registration Committee of the Municipality of Amsterdam and by the Central Committee on Research Involving Human Subjects in the Netherlands.

### Independent variables

Maternal 25OHD concentration was measured at the Regional Laboratory of Amsterdam using an enzyme immunoassay technique (OCTEIA AC-57F1 IDS Ltd, Boldon, UK). The intra and inter-assay coefficients were 8% and <10%, respectively [13]. No results were unreliable per the hemolytic, icteric or lipemic index, but two results were excluded as extreme values. These extreme values were excluded because of the lower reliability of the ELISA test at very high or very low 25OHD levels. Maternal height and pre-pregnancy weight were obtained from the prenatal questionnaire and missing data (3.8% of the data on self-reported height and 9.9% of the data on self-reported weight) were imputed with a random imputation procedure using linear regression analysis [22]. Data on height and weight were then used to calculate pBMI.

### Dependent variables

Continuous heart rate (HR) was measured with the VU University Ambulatory Monitoring System (VU-AMS; Amsterdam, The Netherlands) and blood pressure was measured using an Omron 705 IT device (Omron Healthcare Inc, Bannockburn, IL, USA) after the child was supine for four minutes [23]. Height was measured to the nearest millimeter with a Leicester portable height measure (Seca, Hamburg, Germany) and weight was measured to the nearest 100 g with a Marsden M-4102 scale (Oxforshire, UK) [23]. Offspring BMI was calculated from these measurements. Waist circumference was measured to the nearest millimeter using a Seca measuring tape halfway between the iliac crest and the costal margin and converted into a waist-to-height ratio (WHtR). Percentage body fat (%BF) was measured twice after subjects had emptied their bladders. Measurements were completed in a supine position using tetrapolar bioelectrical impedance analysis (BIA) with the BodyStat 1500 MDD device (BodyStat Inc, Douglas, UK) and the Kushner equation was used to calculate %BF [23, 24]. This technique was previously investigated and validated in four to seven year old children [24].

Offspring blood samples were collected using the Lab Anywhere kit (Haarlem, The Netherlands) and the samples were analyzed in the Regional Laboratory of Amsterdam [25]. Fasting concentrations of total cholesterol (TC) (mmol/L), low-density lipoprotein cholesterol (LDL-C) (mmol/L), high-density lipoprotein cholesterol (HDL-C) (mmol/L), triglycerides (TG) (mmol/L), glucose (mmol/L), and C-peptide (nmol/L) were measured. Because C-peptide was below the detection limit of 0.34 nmol/L in 62% of the subjects, survival analysis was used to impute these values. This was completed in previous research on the ABCD cohort, where a more extensive description of the procedure can be found: in short, imputed C-peptide values were determined on the basis of child age, gender and BMI using a survival analysis method in the statistical package R [25, 26] (R Foundation for Statistical Computing, Vienna, Austria). Insulin resistance (IR) was estimated with a homeostatic model assessment (HOMA2-IR) score calculated from the C-peptide and glucose concentrations [27].

A total of 13 offspring outcome variables were analyzed and because of correlation between these measurements, the outcomes were grouped as follows: cardiovascular function (systolic blood pressure (SBP), diastolic blood pressure (DBP), and HR), adiposity (%BF, WHtR, and BMI), lipid profile (TC, LDL-C, HDL-C, and TG), and glucose metabolism (glucose, C-peptide, and HOMA2-IR).

### Covariates

Covariates were chosen a priori and their measurement has been previously described [13, 23, 25]. In short, covariates were measured using questionnaires that were completed by mothers during pregnancy, as well as when children were 3 months old and when children were 5–6 years old. Chosen covariates included maternal ethnicity (Dutch, non-Dutch), maternal education (years after primary school), parity (nulliparous, multiparous), maternal age during gestation (years), smoking during gestation (yes, no), duration of breastfeeding (none,<1, 1–3, 4–6, or ≥6 months), use of infant vitamin D and/or A-D drops (yes, no), child’s current age (years), child’s gender, and child’s sedentary time (hours). Cardiovascular function outcomes were also adjusted for the child’s current height (cm).

Season and maternal vitamin D supplement use were not adjusted for because they are important determinants of maternal 25OHD status and adjusting for these variables could lead to overcorrection [9, 28]. Maternal co-morbidities (pre- and gestational diabetes mellitus, maternal hypertension, pre-eclampsia and eclampsia), and birth weight were not adjusted for because they are potential mediators [2, 13, 29–31]. Finally, analyses were not corrected for gestational age at sampling: there is conflicting data as to whether 25OHD declines over the course of pregnancy [32–35], and within our study sample, there was no association between gestational age at time of sampling and maternal 25OHD concentration.

### Statistical Analysis

The demographics of the study sample were analyzed across four 25OHD categories: deficient (≤29.9 nmol/L), insufficient (30.0–49.9 nmol/L), and adequate (≥50.0 nmol/L). These categories were chosen for consistency with previous research on the ABCD cohort because the definition of 25OHD deficiency is contentious [13, 36, 37].

Restricted cubic spline (RCS) modelling was used to investigate non-linearity between maternal 25OHD and pBMI, as well as maternal 25OHD and each offspring outcome. The relationship between maternal 25OHD and pBMI was modeled with corrections for maternal covariates (age, education, ethnicity, parity and smoking status). Non-linear models were tested for significant non-linearity with ANOVA and if significant non-linearity was observed, the likelihood ratio test and AIC values were used to determine if the non-linear model fit significantly better than the linear model. If a non-linear relationship was found between 25OHD and a given outcome, RCS modelling was used for statistical testing. If non-linearity was not observed, ordinary least squares linear regression was used for all statistical testing. Model 1 was adjusted for child age and gender (and height for cardiovascular function outcomes). All remaining covariates were added to model 1 to create model 2. To investigate interaction by pBMI in model 2, the likelihood ratio test was used to compare a model with pBMI included as a covariate against a model with pBMI included as an interaction term. PBMI was categorized (underweight (<18.5 kg/m^2^), normal weight (18.5–24.9 kg/m^2^), and overweight (≥25.0 kg/m^2^)) to enhance interpretability. A p-value <0.1 was considered a significant difference between the two models because this is the standard p-value cut-off when investigating interaction to avoid missing a potentially important interacting variable. The better fitting model was determined by comparing the AIC values. Finally, a two-sided Wald test was used to determine if the interaction terms were significant for underweight women compared to normal weight women and for overweight women compared to normal weight women. If no interaction was observed, pBMI was not added to the models as a covariate because pBMI is likely a determinant of maternal 25OHD [38] and its addition to the models could lead to overcorrection.

Post-hoc analyses were conducted to determine if the results would change substantially with adjustment for season and maternal vitamin D supplement use, analysis of the Dutch-only sample, and analysis of the sample excluding women with co-morbidities. Analyses of offspring C-peptide and HOMA2-IR were also repeated excluding the imputed C-peptide data.

A Bonferroni correction for multiple outcomes was applied based on the four correlated outcome groups, which resulted in a p-value <0.0125 as significant (0.05 divided by 4). Therefore, a p-value <0.01 was treated as significant for simplicity. Normality of the outcome variables was investigated and appropriate transformations were determined via the Box-Cox method. Both transformed and untransformed data were analyzed to ensure statistical robustness, while allowing presentation of the untransformed model. Data missing from the covariates included in model 2 were imputed using multivariate imputation by chained equations (MICE) in R, which utilizes all covariates in the dataset to generate values for missing data [39]. 5 datasets were generated using MICE and all statistical testing was based on the average values of these 5 datasets. SPSS version 20 (SPSS Inc, Armonk, NY, USA) and R version 2.13.1 (R Foundation for Statistical Computing, Vienna, Austria) were used for all statistical analyses.

## Results

**Fig 2:**
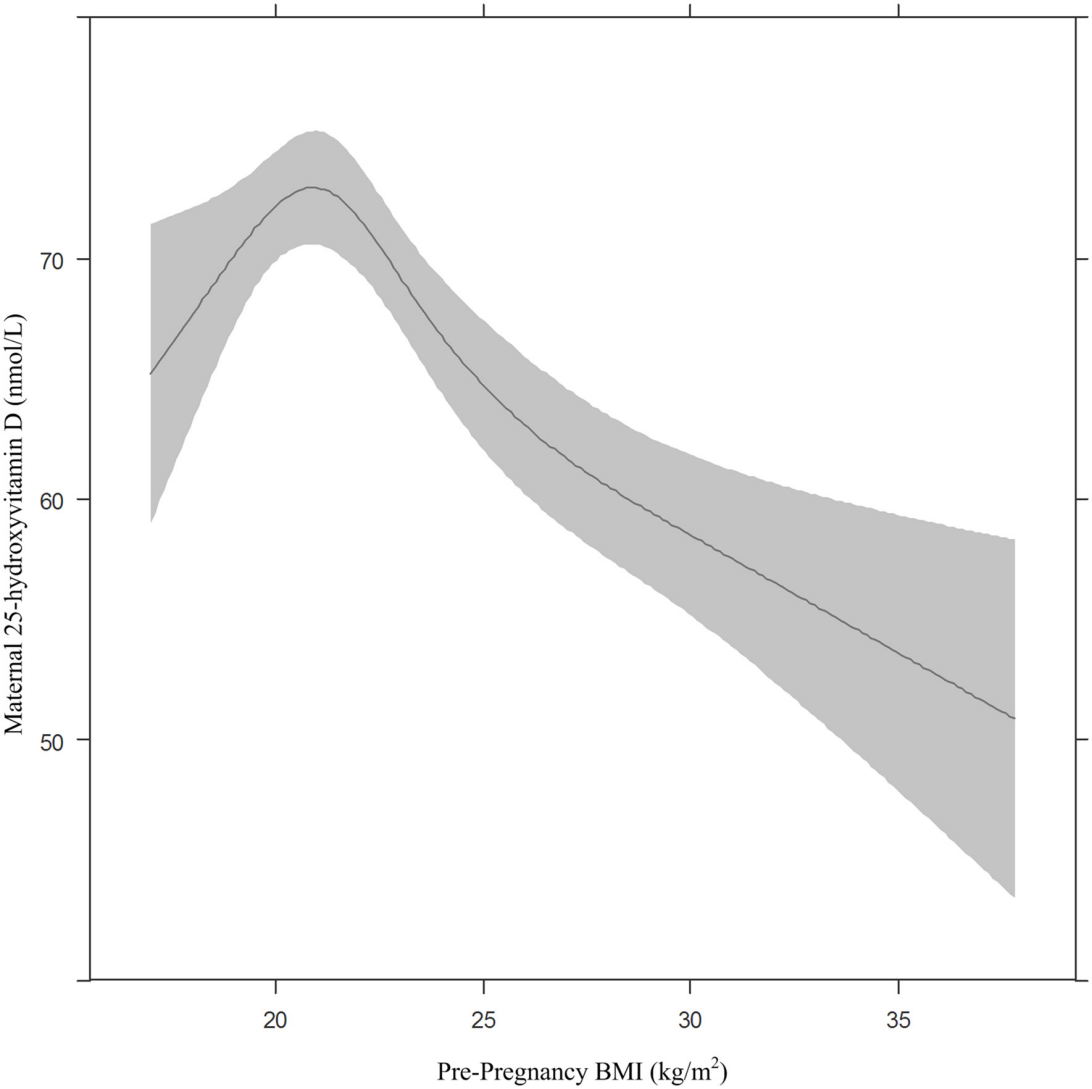
The association between maternal 25-hydroxyvitamin D concentration in early pregnancy and pre-pregnancy BMI. The association between maternal 25-hydroxyvitamin D and pre-pregnancy BMI modeled using restricted cubic splines. Adjusted for maternal age (31.9 years) and maternal education (10 years) and presented for Dutch ethnicity, nulliparous women and non-smokers

2150 eligible mother-child pairs did not complete follow-up as a result of non-response, leaving Amsterdam or death. The mothers in this non-response group were younger, less educated, more often non-Dutch, and were more likely to have smoked during pregnancy (Table 1). Of those who did complete follow-up, 17.7% of the women had deficient 25OHD levels and 20.7% had insufficient levels (Table 2). The mean maternal 25OHD concentration in the study sample was 60.4 nmol/L (interquartile range, 18.9–102.7 nmol/L), which was measured at a mean gestational age of 16.1 weeks. Maternal 25OHD deficiency was more likely if blood samples were drawn in winter, pBMI was higher, and women were younger, less educated, not Dutch, multiparous, smokers during pregnancy, and not users of vitamin D supplements. The children of deficient mothers were generally older and more sedentary. 25OHD deficiency was more prevalent in both underweight (26.4%) and overweight (31.5%) women when compared to normal weight women (13.6%), and the relationship between pBMI and 25OHD was significantly non-linear (p = 0.002) (Fig 2). In the study sample there were 1426 normal weight, 72 underweight and 384 overweight women.

**Table 1:** Comparison of eligible mothers whose children were suitable for follow-up who either completed (responders) or did not complete follow-up (non-responders).

Variable	N	Responders (N = 1882)		Non-responders (N = 2150)		P-value†
		Mean	SD	Mean	SD	
**25OHD**	4032	60.4	0.7	52.4	0.7	<0.001
**pBMI**	4032	22.9	3.7	22.9	3.8	0.8
**Age (years)**	4032	31.9	4.4	30.2	5.1	<0.001
**Education (years after primary school)**	4001	10.0	3.6	8.5	3.9	<0.001
**Ethnicity (% Dutch)**	4024	69.0		50.5		<0.001
**Parity (% nulliparous)**	4032	57.5		57.5		1.0
**Smoking during gestation (% smoker)**	4032	8.3		10.4		0.02

25OHD = 25-hydroxyvitamin D, pBMI = pre-pregnancy BMI, N = number of women with data available on a given covariate

^†^P-value for difference between responders and non-responders, as tested by X^2^ for categorical variables and independent sample t-tests for continuous variables

**Table 2:** Study population characteristics as a function of maternal 25-hydroxyvitamin D status in early pregnancy.

Variable	N	Maternal 25-Hydroxyvitamin D Status								P-Value†
		All		Deficient (≤29·9nmol/L)		Insufficient (30·0-49·9 nmol/L)		Adequate (≥50·0 nmol/L) ‡		
		Mean	SD	Mean	SD	Mean	SD	Mean	SD	
**Study sample (N)(%)**	1882			334 (17.7)		390 (20.7)		1158 (61.5)		
***Maternal Factors***										
**Season of 25 OHD sample (%)**	1875									<0.001
-Summer		24.4		10.8***		11.6***		32.7		
-Fall		19.9		14.1		18.6		22.1		
-Winter		36.9		59.9		49.7		25.8		
-Spring		18.8		15.3		20.1		19.3		
**pBMI (kg/m** ^**2**^ **)**	1882	22.9	3.7	24.2***	4.9	23.6***	4.3	22.2	2.9	<0.001
**Age (years)**	1882	31.9	4.4	29.7***	5.5	32.0	4.4	32.4	3.7	<0.001
**Education (years after primary school)**	1875	10.0	3.6	7.4***	4.3	9.9***	3.6	10.8	2.9	<0.001
**Ethnicity (%)**	1882									<0.001
-Dutch	1299	69.0		29.6***		64.4***		82.0		
-African-descent	74	3.9		9.6		6.2		1.6		
-Turkish	50	2.7		11.4		2.1		0.3		
-Moroccan	89	4.7		19.8		3.8		0.7		
-Other western	234	12.4		7.5		14.1		13.3		
-Other non-western	136	7.2		22.2		9.5		2.2		
**Parity (% nulliparous)**	1882 1082	57.5		47.9***		56.7		60.5		<0.001
**Smoking (% Non-smoker)**	1882 1726	91.7		88.3**		89.7*		93.4		0.004
**Vitamin D supplement use (% yes)**	1643 640	39.0		25.8***		32.1***		44.2		<0.001
***Child Factors***										
**Gender (% male)**	1882 918	48.8		48.2		49.5		48.7		0.9
**Age (years)**	1874	5.7	0.5	5.9***	0.5	5.7*	0.5	5.7	0.5	<0.001
**Height (cm)**	1871	116.7	5.6	117.2	5.8	116.7	5.6	116.5	5.6	0.2
**Duration breastfeeding (%)**	1859									<0.001
-none	301	16.2		18.2***		19.2		14.6		
-<1 month	122	6.6		11.6		6.2		5.2		
-1-3 months	497	26.7		28.6		23.6		27.2		
-3-6 months	599	32.2		22.2		32.7		34.9		
-≥6 months	340	18.3		19.5		18.2		18.0		
**Use of vitamin drops (% no)**	1664 174	10.5		11.8		12.7		9.4		0.2
**Sedentary time (hours)**	1731	1.4	1.0	2.1***	1.3	1.5***	1.1	1.2	0.7	<0.001

25OHD = 25-hydroxyvitamin D, pBMI = pre-pregnancy BMI

Mean values significantly different from reference:

*p<0.05,

**p<0.01,

***p<0.001

^†^P-value for differences between vitamin D categories, as tested by X^2^ for categorical variables, and ANOVA and independent sample t-tests for continuous variables

^‡^Reference group

**Fig 3:**
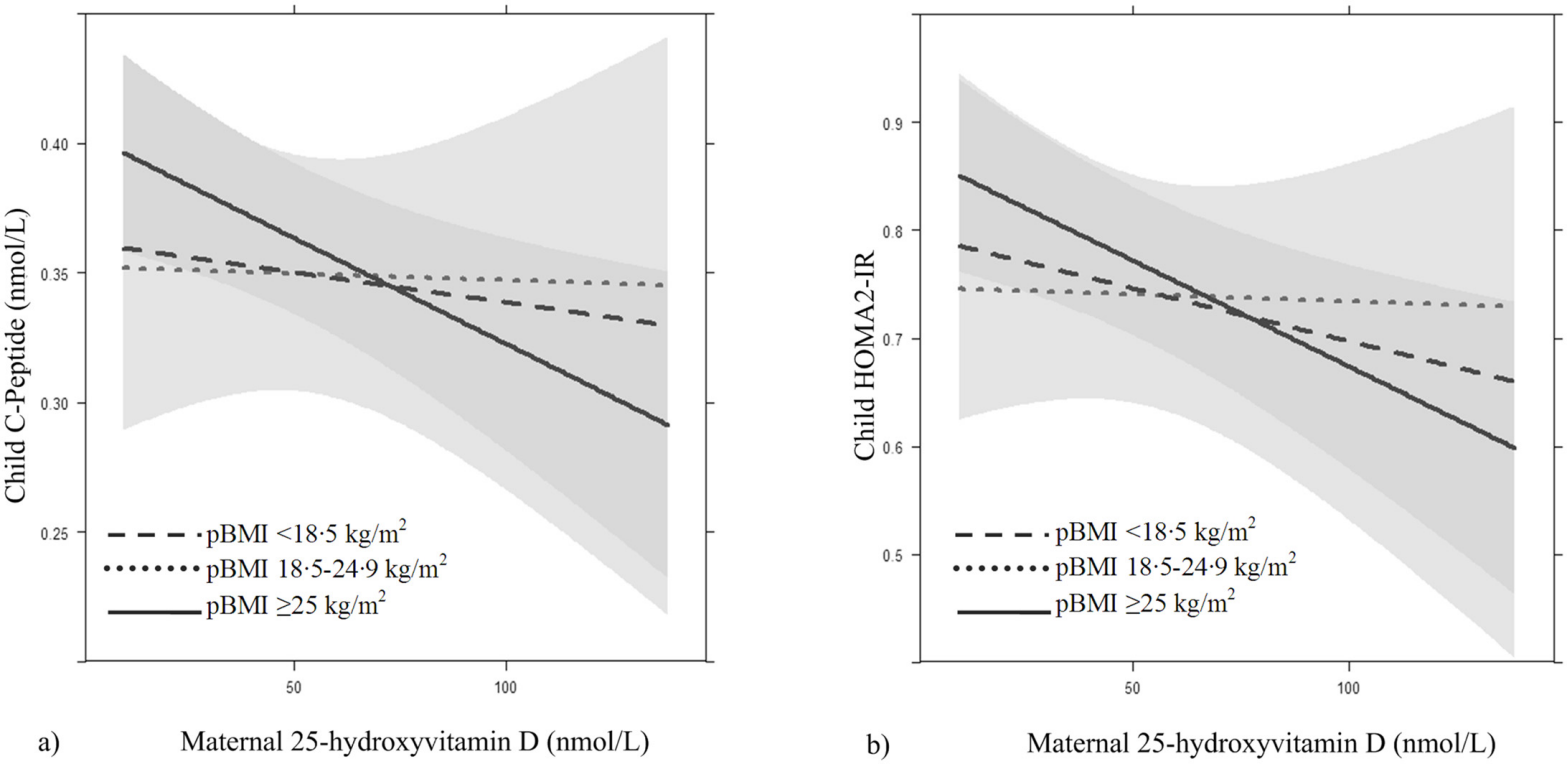
The association between maternal 25-hydroxyvitamin D concentration and insulin resistance markers in young children. The association between maternal 25-hydroxyvitamin D concentration in early pregnancy and a) C-peptide and b) HOMA2-IR in 5–6 year old children with interaction with pre-pregnancy BMI presented. Adjusted for maternal age (32.4 years), maternal education (10 years), child age (5.6 years) and sedentary time (1.4 hours), and presented for Dutch ethnicity, nulliparous mothers, non-smokers, male children, no use of vitamin D or A/D drops in infancy and no history of breastfeeding.

None of the associations between maternal 25OHD and child cardio-metabolic outcomes were significantly non-linear with the exception of the association with child %BF (Table 3). Interaction with pBMI was observed for the associations with C-peptide (p = 0.02) and HOMA2-IR (p = 0.02); the models with interaction terms fit significantly better than the models without interaction terms on basis of the likelihood ratio test and AIC values. Significant inverse associations between maternal 25OHD and each of these outcomes were observed in children born to overweight women. For this group, a 10 nmol/L increase in maternal 25OHD was significantly associated with a 0.007 nmol/L decrease in offspring C-peptide (99%CI:-0.01,-0.001) and a 0.02 decrease in offspring HOMA2-IR (99%CI:-0.03,-0.004) (Fig 3). No significant associations between maternal 25OHD and these two outcomes were found in the children of underweight or normal weight women.

**Table 3:** Linear regression models of the association between maternal 25-hydroxyvitamin D concentration in early pregnancy and cardio-metabolic outcomes in 5–6 year old children (per 10 nmol/L increase of 25-hydroxyvitamin D) with testing for interaction with pre-pregnancy BMI.

Outcome	N	Mean of outcome variable (SD)		Model 1^1^		Model 2^2^	
				ß	99%CI	ß	99%CI
***Cardio-vascular Function***							
**SBP (mmHg)**	1841	99.6	(8.2)	-0.1	(-0.3, 0.01)	-0.06	(-0.2, 0.09)
**DBP (mmHg)**	1834	57.5	(6.8)	-0.2	(-0.3, -0.09)	-0.08	(-0.2, 0.05)
**HR (bpm)**	1668	85.6	(10.8)	-0.2	(-0.4, 0.008)	-0.1	(-0.3, 0.1)
***Adiposity***							
**BMI (kg/m** ^**2**^ **)**	1870	15.6	(1.7)	-0.05	(-0.08, -0.02)	-0.02	(-0.06, 0.007)
**Body fat (%)**	1843	20.8	(6.9)	*	-	**-0.1**	**(-0.3, -0.003)**
**WHtR**	1869	0.45	(0.04)	-0.001	(-0.002,-0.0004)	-0.0006	(-0.001, 0.00007)
***Lipid Profile***							
**TC (mmol/L)**	1201	4.0	(0.8)	0.01	(-0.005, 0.03)	0.02	(-0.002, 0.04)
**LDL-C (mmol/L)**	1201	2.3	(0.8)	0.01	(-0.006, 0.03)	0.01	(-0.005, 0.03)
**HDL-C (mmol/L)**	1201	1.3	(0.4)	-0.0005	(-0.008, 0.007)	-0.0002	(-0.009, 0.009)
**TG (mmol/L)**	1201	0.65	(0.4)	-0.002	(-0.009, 0.006)	-0.0001	(-0.009, 0.009)
***Glucose Metabolism***							
**Glucose (mmol/L)**	1201	4.6	(0.6)	-0.002	(-0.01, 0.01)	0.0004	(-0.01, 0.01)
**C-peptide (nmol/L)**	898	0.35	(0.1)	†	-	†	-
**HOMA2-IR**	898	0.75	(0.3)	†	-	†	-

99%CI = 99% confidence interval, bpm = beats per minute, DBP = diastolic blood pressure, HOMA2-IR = homeostatic model assessment of insulin resistance, HDL-C = high density lipoprotein cholesterol, HR = heart rate, LDL-C = low density lipoprotein cholesterol, SBP = systolic blood pressure, TC = total cholesterol, TG = triglyceride, WHtR = waist-to-height ratio

^1^adjusted for child age and gender (and child height for cardiovascular function outcomes);

^2^additionally adjusted for maternal education, ethnicity, maternal age, parity, smoking during gestation, duration of breastfeeding, use of vitamin D or A/D drops in infancy and child’s sedentary time

**Fig 4:**
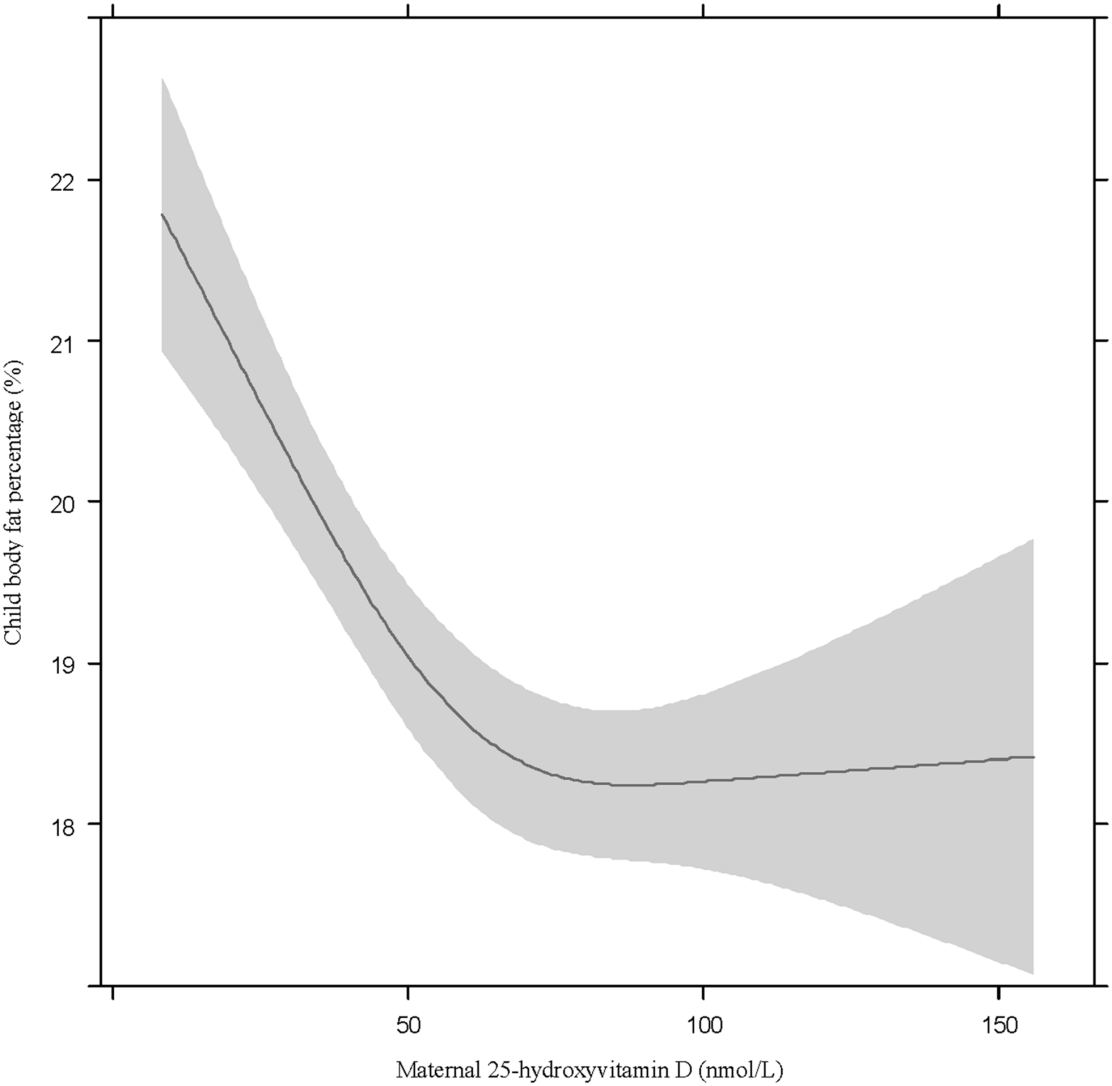
The association between maternal 25-hydroxyvitamin D concentration and percentage body fat in young children. The association between maternal 25-hydroxyvitamin D concentration and percentage body fat in 5–6 year old children, per model 1 (adjusted for child age (5.7 years) and presented for male children).

*Significant non-linear relationship presented in Fig 4

^†^ Significant interaction. Associations presented in Fig 3

No interactions were observed for the remaining cardio-metabolic outcomes. In model 1, a non-linear, inverse association between maternal 25OHD and %BF was observed, which plateaued at ~75 nmol/L of 25OHD (Fig 4). After correction (model 2), the association between maternal 25OHD and child %BF was no longer significantly non-linear, but there was a significant 0.13% decrease in %BF (99%CI: -0.3, -0.003) per 10 nmol/L increase in maternal 25OHD. Additionally in model 1, a 10 nmol/L increase in maternal 25OHD was significantly associated with a 0.21 mmHg lower DBP (99%CI: -0.3, -0.09), a 0.05 kg/m^2^ lower BMI (99%CI: -0.08, -0.02), and a 0.001 lower WHtR (99%CI: -0.002, -0.0004) in the children, but these relationships were eliminated after adjustment for confounding (model 2): ethnicity and sedentary time were responsible for the largest attenuation in the effect sizes.

When the analyses were repeated excluding women with co-morbidities (N = 1635), in the Dutch only group (N = 1299) or when adjusted for season and vitamin D supplement use, the association between maternal 25OHD and %BF in model 2 lost significance. Despite this, the upper limit of the 99% CI just crossed 0 in each of these sensitivity analyses, and the effect sizes observed in these sensitivity analyses were only slightly attenuated in comparison to the overall analysis (exclusion of co-morbidities: β:-0.13, 99%CI:-0.3, 0.006; exclusion of non-Dutch ethnicity: β:-0.10, 99%CI:-0.3, 0.005; adjustment for season and supplement use: β:-0.10, 99%CI:-0.3, 0.03). Finally, in the sensitivity analysis of non-imputed C-peptide data (N = 322), the models with pBMI included as an interaction term did not fit significantly better than the models with pBMI included as a covariate. However, the Wald test suggested a trend towards interaction for overweight women compared to normal weight women (p = 0.2 for C-peptide and p = 0.14 for HOMA2-IR).

## Discussion

This study observed inverse associations between maternal 25OHD and the markers of IR (C-peptide and HOMA2-IR) in children whose mothers were overweight during pregnancy. Additionally, an inverse relationship was observed between maternal 25OHD and offspring %BF, but no interaction with pBMI was observed in this association or for any other outcomes. A non-linear association between pBMI and maternal 25OHD was also observed.

### Strengths and limitations

Although these results are promising, strong conclusions cannot be drawn at this time because the main findings did not hold in the non-imputed C-peptide data. Data were imputed using the best possible method available, via survival analysis, on the basis of child age, gender and BMI. Additionally, the range of C-peptide values in the non-imputed data was narrower due to the exclusion of all samples below 0.34 nmol/L which reduced the possibility of detecting an association in this sensitivity analysis. Finally, there were trends for significant interaction in the non-imputed data despite the much smaller sample size. However, strong conclusions can only be drawn from future research on this topic by using more sensitive C-peptide assays. Ultimately, it is possible that young children suffer from increased insulin resistance when exposed to both maternal overweight and low 25OHD in utero, but future studies are necessary to confirm this.

The association between maternal 25OHD and child %BF also lost significance in the sensitivity analyses. Therefore, it is not possible to rule out the presence of residual confounding in the main analysis. However, the loss of significance could be the result of overcorrection in the sensitivity analysis that included season and vitamin D supplement use because these two variables are determinants of vitamin D status. In the Dutch-only sensitivity analysis and the sensitivity analysis excluding maternal co-morbidities, the lower extremes of maternal 25OHD and the higher extremes of child %BF were excluded, which reduced the chance of detecting a significant relationship. Additionally, the association was borderline non-significant and the effect sizes were not dramatically altered from the main analysis, suggesting a loss of power may be responsible for these results. Ultimately, the findings of the primary analysis are supported by previous research (4, 9), however, additional studies investigating the mediating properties of season, supplement use and maternal co-morbidities in the relationship, as well as more studies in non-Western ethnic groups are necessary to confirm the results of the current study.

The current study was also limited by selective drop-out because mothers whose children completed follow-up had higher SES, were more often Dutch, and were less likely to have smoked during pregnancy. Higher risk individuals were lost to follow-up, which may have biased the results and possibly limited the strength of the observed effect sizes. Therefore additional research on this topic in higher risk populations is also necessary.

The current study benefited from a large sample size, corrections for testing multiple outcomes and testing of non-linearity. 25OHD concentration was also measured early in pregnancy which is a critical period for the establishment of maternal tolerance to the embryo and placenta [40]. Finally, we believe this was the first study to investigate interaction with pBMI in the relationship between maternal 25OHD and child cardio-metabolic outcomes.

### Potential biological mechanisms

A non-linear relationship between maternal 25OHD and pBMI was clearly observed in the current study. The proportions of women with deficient 25OHD levels in early gestation were greater in both the underweight and overweight women, suggesting that the observed parabolic association was not simply a statistical artifact of the small number of underweight women. Additionally, the observed non-linear relationship corresponds with previous research in pregnant and non-pregnant subjects [12, 13, 19]. In overweight individuals, dietary intake and sun exposure may partially influence 25OHD levels, but sequestration in adipose tissue appears to be the major mechanism [38, 41]. The mechanism in underweight women is less clear due to limited research in this population. It is possible that more 25OHD remains in the bloodstream as a result of a lower fat mass, leading to faster elimination [12]. Alternatively, deficiency in this population could result from poor dietary intake of vitamin D [42].

Although higher pBMIs are associated with lower maternal 25OHD in early gestation [13, 19], several studies have found no associations between pBMI and 25OHD in late gestation [9, 11, 20]. The reason for this inconsistency is unclear. Perhaps over the course of pregnancy, normal weight women gain proportionally more adipose tissue than overweight women, resulting in comparable sequestration of 25OHD [17]. Despite this, fetuses receive less 25OHD from overweight women [19, 20], perhaps due to 25OHD sequestration in adipose tissue early in pregnancy and impaired 25OHD transfer later in pregnancy. In human and animals models of obesity during pregnancy, alterations in placental structure as well as increased placental inflammation have been observed, both of which could contribute to placental dysfunction [16, 43]. Dysregulation of vitamin D metabolism has also been associated with alterations in placental inflammatory pathways [40, 44] and vitamin D deficiency in rat dams has been associated with abnormal placental vascularization [44]. Thus it is possible that an elevated pBMI leads to both placental dysfunction and maternal 25OHD deficiency, which in itself contributes to placental dysfunction, ultimately impairing 25OHD transfer to the fetus. However, more research is necessary to fully understand the mechanisms involved.

The inverse associations observed between maternal 25OHD and IR markers in children of overweight women are logical when considering the current evidence. When studied separately, both 25OHD and maternal overweight have been related to diabetes mellitus. Overweight during pregnancy appears to increase the risk for type 2 diabetes mellitus in offspring [16–18] by contributing to placental inflammation and causing excessive nutrient transfer to the fetus [16]. 25OHD deficiency within individuals is associated with an increased risk of type 2 diabetes mellitus [45], possibly via influence on beta cell function and insulin sensitivity [5, 46]. Additionally, recent work in rats has shown that vitamin D deficient dams give birth to insulin resistant pups and that this IR worsens as the pups age [7]. The results of this study appear to be related to epigenetic mechanisms that are mediated by maternal 25OHD and which subsequently promote inflammation in the offspring [7]. Perhaps in humans the combination of low maternal 25OHD and overweight are sufficient to alter glucose metabolism in offspring leading to the development of IR.

Similar relationships between maternal 25OHD and IR in the children of underweight mothers were not observed, but a slight trend could be seen on graphical analysis (Fig 3). If an inverse association between maternal 25OHD and offspring IR exists for this group, the mechanisms may involve restricted fetal growth. Both low maternal 25OHD and maternal underweight have been associated with lower birth weight [13, 14] and lower birth weight has been associated with an increased risk of type 2 diabetes mellitus [1, 47]. Unfortunately, little can be concluded about this group of mothers and children because of the limited research.

The inverse association observed between maternal 25OHD and child %BF is also consistent with previous observations [4, 9]. There is some evidence that the 25OHD metabolite, 1,25-dihydroxyvitamin D, may directly influence offspring lipid storage and metabolism, and 25OHD may also be involved in normal offspring muscle development, which could influence the proportion of fat- to fat-free mass [48]. No interaction with pBMI was observed for this association, suggesting that lower 25OHD is associated with increases in offspring body fat but that the combined exposure of low 25OHD and abnormal pBMI does not result in even higher offspring adiposity. Perhaps 25OHD functions as a mediator in the relationship between pBMI and offspring body fat, however further investigations are necessary to confirm this hypothesis.

### Previous research

The results of this study may help to clarify inconsistencies from previous research [4, 9–11]. Williams et al. [11] did not observe an inverse association between maternal 25OHD and offspring IR markers, while Krishnaveni et al. did [4]. Perhaps differences in pBMIs and the ethnic compositions of these two cohorts contributed to the disparate findings. Williams et al. [11] also observed an inverse association between maternal 25OHD and SBP in ten year old children, but the finding was not highly significant, nor was it observed in the current research or in other studies [4, 10]. Thus, the possibility of type I error cannot be ruled out. Our observation of an inverse relationship between maternal 25OHD and child %BF was similar to the results from Crozier et al. and Krishnaveni et al., but contrary to those from Gale et al. [4, 9, 10]. It is possible, however, that Gale et al. did not detect this association due to the small sample size (N = 178) [10]. Finally, the lack of associations with the remaining cardio-metabolic outcomes in the current study appears to be in line with previous research [4, 9–11] indicating that if maternal 25OHD affects cardio-metabolic health of offspring, the effects are limited to body composition and glucose metabolism.

### Implications

As stated previously, strong conclusions cannot be drawn from these results due to the limitations of the undetectable C-peptide data. However, future research in overweight women may help to confirm whether an inverse relationship between maternal 25OHD and offspring IR exists for this population. Additionally, while limited by possible residual confounding, the observed association between maternal 25OHD and offspring adiposity has been observed in the previous literature [4, 9]. Perhaps offspring %BF could be investigated as an outcome in some of the many vitamin D supplementation trials in pregnant women that are currently underway [49] to help clarify if there is indeed a causal relationship between maternal 25OHD and child adiposity.

## Conclusions

The results of this study suggest that low maternal 25OHD is associated with increased IR in the children of overweight women, and that low maternal 25OHD is associated with increased %BF in children with no interactive effects from pBMI. Due to the limitations of this study, these results are not conclusive, however the observations of this study pose important research questions for future studies to investigate.
